# Acute Colonic Pseudo-Obstruction: A Case of Ogilvie Syndrome

**DOI:** 10.7759/cureus.66038

**Published:** 2024-08-02

**Authors:** Carissa Du, Nofel Iftikhar, Latha Ganti, April Smith-Gonzalez

**Affiliations:** 1 Biology, Buchholz High School, Gainesville, USA; 2 Biology, University of Florida, Gainesville, USA; 3 Emergency Medicine and Neurology, University of Central Florida, Orlando, USA; 4 Research, Orlando College of Osteopathic Medicine, Winter Garden, USA; 5 Medical Sciences, The Warren Alpert Medical School of Brown University, Providence, USA; 6 Osteopathic Manipulative Medicine, Orlando College of Osteopathic Medicine, Winter Garden, USA

**Keywords:** ct-scan, bowel conservation, cesarean section (cs), adult gastroenterology, ogilvie's syndrome

## Abstract

Ogilvie syndrome is a pseudo-obstruction of the large colon that does not contain any mechanical obstruction. This is a case of a 32-year-old female who presented to the emergency department (ED) for acute onset vomiting and intermittent watery diarrhea. Based on her presentation, physical exam, and computed tomography (CT) findings, she was diagnosed with Ogilvie syndrome. The pathophysiology of Ogilvie syndrome involves a disruption of normal colonic motility, leading to excessive gas and fluid accumulation. Abdominal imaging typically reveals a massively dilated colon with no evident obstructive lesion. Initial management is conservative and includes supportive measures such as bowel rest and decompression, and may include medications to enhance colonic motility. In severe cases, endoscopic or surgical intervention to relieve symptoms and prevent complications such as bowel ischemia or perforation may be necessary. Identifying and addressing underlying precipitating factors is crucial for effective treatment and preventing recurrence.

## Introduction

Ogilvie syndrome is characterized by an intestinal pseudo-obstruction of the large colon in the absence of any mechanical obstacles. This obstruction prevents the contraction of the intestines, restricting regular movement of stool, food, and air through the digestive tract [1-2]. In most cases of Ogilvie syndrome, the dilation of the bowel is limited to the cecum, ascending colon, and splenic flexure. Ogilvie syndrome is a relatively uncommon incidence, only composing of 0.1% (100/100,000) of all inpatient admissions annually [3]. Patients with Ogilvie syndrome present symptoms of bowel obstruction, nausea, vomiting, abdominal distension, and obstipation with bowel obstruction on CT imaging or x-ray [4-5]. Left untreated, Ogilvie syndrome may cause colon perforation, which features an overall mortality rate of 40%, and ischemia, which may lead to colitis or necrosis [4]. These serious complications make Ogilvie syndrome a dangerous disease with life-threatening complications [6-8]. In this case report, the authors describe a case of Ogilvie syndrome and the treatment provided to the patient.

## Case presentation

This is the case of a 32-year-old female who presented to the emergency department (ED) due to acute onset vomiting. The patient experienced nausea and intermittent watery diarrhea, which was reported to be free of blood. The patient’s surgical history included a cesarean section (C-section) and tubal ligation. Neither the patient’s husband nor daughter experienced any similar symptoms and the patient denied eating any non-homecooked food prior to the onset of symptoms. The patient reported being a heavy smoker, which may have played a role in disease manifestation due to immunocompromisation. The patient’s vitals were as follows: O_2_ saturation 99% on room air, blood pressure 104/60 mmHg, temperature 99.5° F, pulse 95 beats per minute, and a respiratory rate of 16 breaths per minute.

**Figure 1:**
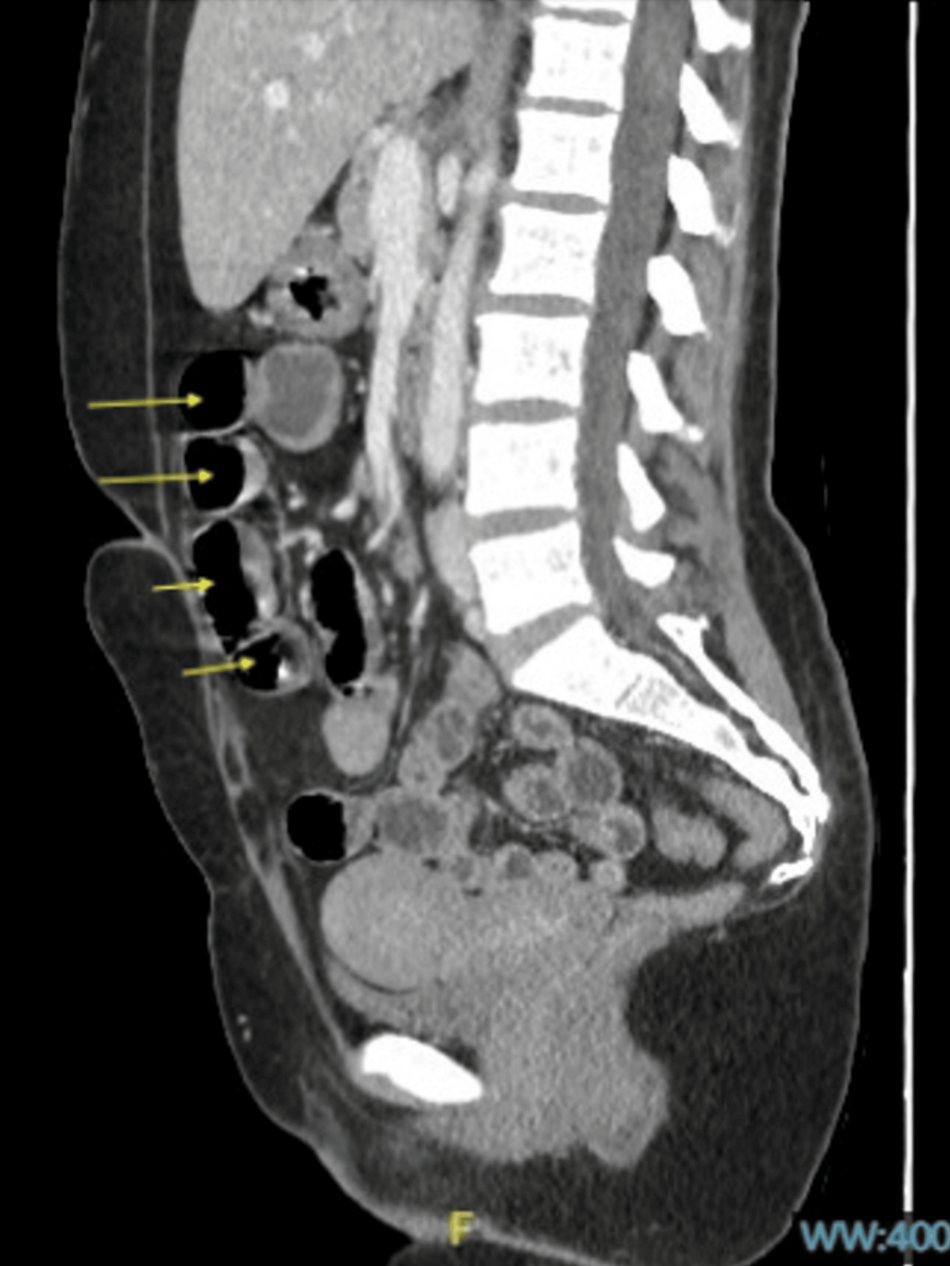
Abdominal CT scan demonstrating colonic pseudo-obstruction denoted by yellow arrows.

**Figure 2:**
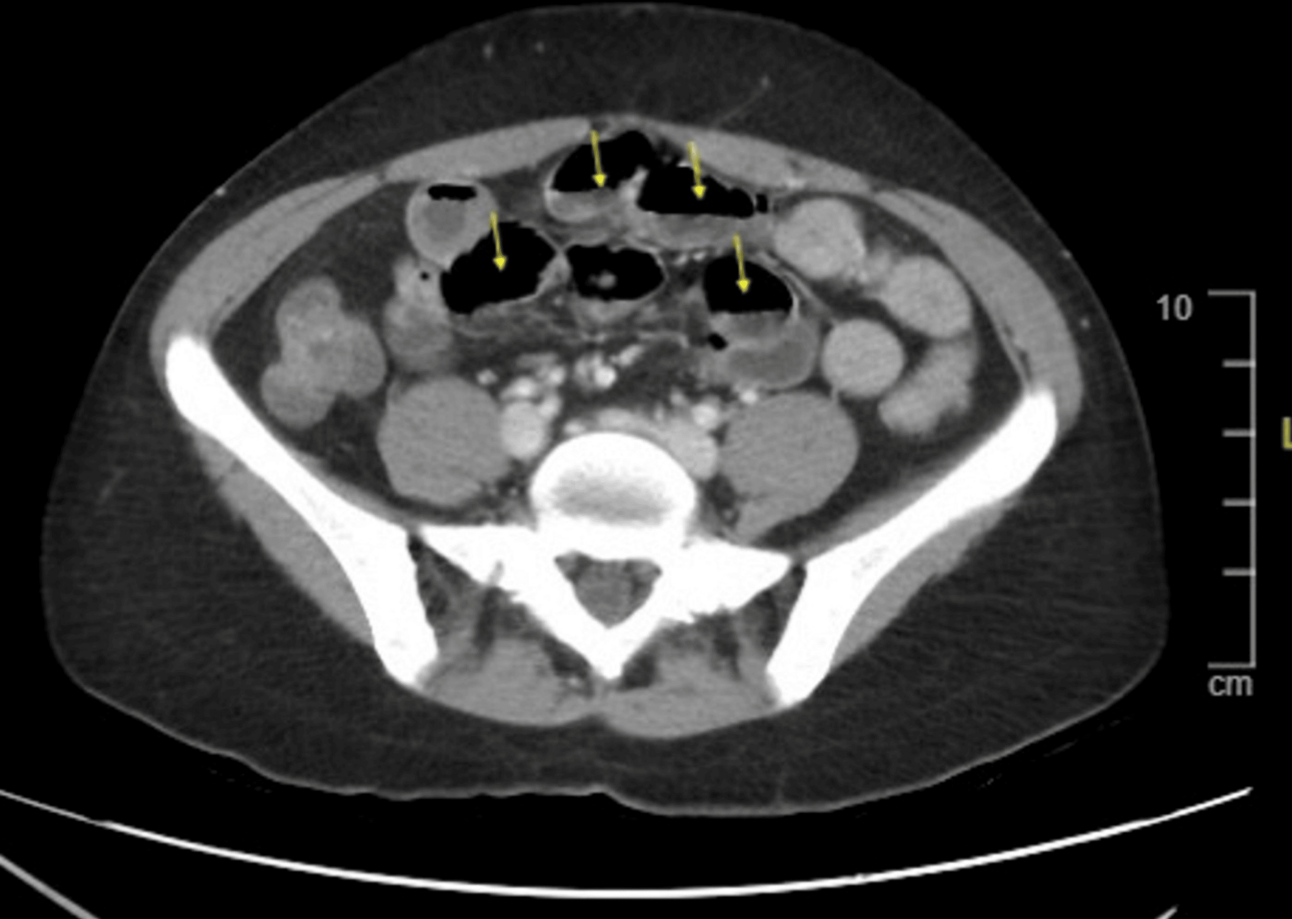
Axial view abdominal CT scan demonstrating fluid and air-filled loops of bowel denoted by yellow arrows.

Laboratory analysis revealed leukocytosis with a white blood cell count of 22000 x 10^9^/L, which warranted the usage of intravenous (IV) piperacillin-tazobactam, and a CT scan. Additionally, to mitigate symptoms of nausea and pain, the patient received 10 mg of IV metoclopramide, 15 mg of IV ketorolac, and one liter of IV normal saline solution. CT of the abdomen and pelvis revealed the colon to have dilated loops of bowel with air-fluid level extending from the cecum to the splenic flexure with a transition point. Also noted was a hiatal hernia. There were no ascites, abdominal mass, pathologic lymphadenopathy, or evidence of small bowel obstruction. These findings confirmed a diagnosis of Ogilvie syndrome (Figures 1, 2).

The patient was admitted to the hospital for further observation and to continue antibiotic treatment. After three days of treatment, the patient was successfully cleared for home discharge.

## Discussion

Patients with a dilated colon, as a result of Ogilvie syndrome, are at risk of toxic megacolon, a life-threatening condition, which results in deep colon swelling and inflammation [9-10]. Additionally, Ogilvie syndrome can result in several electrolyte disorders, including hypokalemia, hypomagnesemia, and hypocalcemia [11]. Ogilvie syndrome often presents following a trigger such as immobile lifestyle, specific medications which can slow intestinal movements, bouts with cardiovascular disease or infection, pre-existing electrolyte imbalances, and neurological diseases. Though these factors may put an individual at a higher risk of developing Ogilvie syndrome, its etiology is undefined and unpredictable. Ogilvie syndrome is most prevalent in elderly populations, most readily explained by their relatively immobile lifestyle and pre-existing health conditions [12].

In the case presented, the patient’s surgical history, C-section and ligation, may also indicate possible risks which lead to the development of Ogilvie syndrome [13]. The C-section, in particular, was of note being one of the surgeries most associated with the development of Ogilvie syndrome. Post-C-section Ogilvie syndrome has been seen to have a relatively high mortality rate due to cecal perforation, which is most common in cases diagnosed late [14-15]. However, in the presenting case, the patient was diagnosed relatively early and was able to make a full recovery using conservative treatment methods, which included antibiotics, fluids, rest, and regular monitoring.

Treatment for Ogilvie syndrome is most commonly dictated by the severity of cecum deviation. If it is observed that the cecum presents a diameter of nine centimeters or greater, surgical intervention is necessary (puncture decompression or cecostomy). If the cecum deviation is nine centimeters or less, as was the case in the report presented, treatment consists of antibiotics, fluids, rest, and monitoring [4].

**Figure 3:**
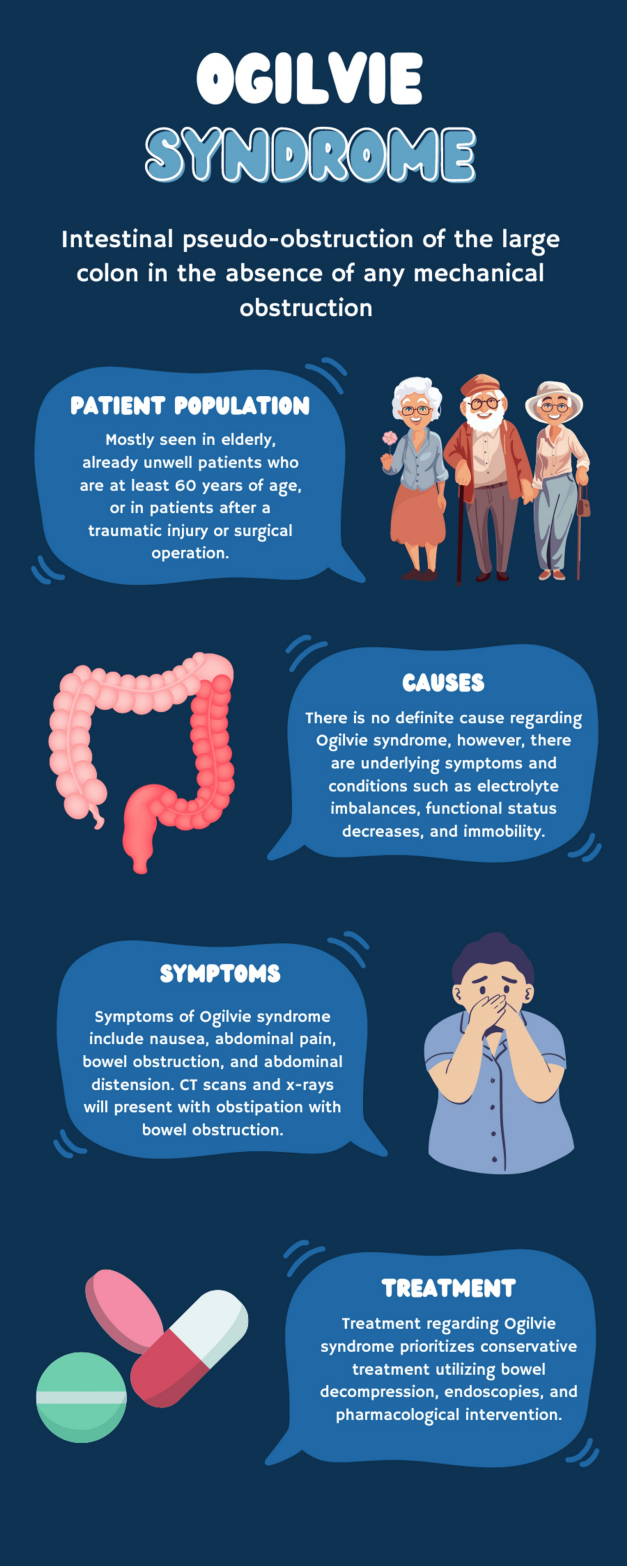
Infographic featuring description, symptoms, presentation, and treatment of Ogilvie syndrome. Infographic designed by Carissa Du.

Additionally, immediate bowel decompression is a primary focus in treating Ogilvie syndrome. This is most readily performed via an escalating model of treatment beginning with a nasogastric tube and diet restriction. If the patient shows no signs of improvement following 72 hours, pharmacological intervention is required, and if no improvement is still observed, endoscopic therapies are performed. Further surgical intervention is only needed if the endoscopic treatments are not proven effective or if the patient develops ischemia or perforations [3]. Traditional management for Ogilvie syndrome dictates that conservative treatment should be considered first without moving on to pharmacological interventions and endoscopic therapies (Figure 3) [16].

## Conclusions

Ogilvie syndrome is a dilation of the colon from the cecum to the splenic flexure with no mechanical obstruction. In the presenting case, the patient’s CT imaging and symptomatic conditions both displayed typical signs of Ogilvie syndrome. Her surgical history, consisting of a C-section and ligation, were seen to be contributing factors to the onset of the diagnosis. The patient was treated conservatively with antibiotics, fluids, and hospitalization monitoring, and made a full recovery.
